# Detecting and monitoring bladder cancer with exfoliated cells in urine

**DOI:** 10.3389/fonc.2022.986692

**Published:** 2022-09-07

**Authors:** Nannan Li, Lei Wang, Han Liang, Cong Lin, Ji Yi, Qin Yang, Huijuan Luo, Tian Luo, Liwei Zhang, Xiaojian Li, Kui Wu, Fuqiang Li, Ningchen Li

**Affiliations:** ^1^ College of Life Sciences, University of Chinese Academy of Sciences, Beijing, China; ^2^ Beijing Genomics Institute (BGI)-Shenzhen, Shenzhen, China; ^3^ Guangdong Provincial Key Laboratory of Human Disease Genomics, Shenzhen Key Laboratory of Genomics, Beijing Genomics Institute (BGI)-Shenzhen, Shenzhen, China; ^4^ Department of Urology, Peking University Shougang Hospital, Beijing, China; ^5^ Peking University Wu-jieping Urology Center, Peking University Health Science Center, Beijing, China

**Keywords:** bladder cancer, urine, LHC-BS, noninvasive, DNA methylation arctangent score (DMAS)

## Abstract

Current methods for the diagnosis and monitoring of bladder cancer are invasive and have suboptimal sensitivity. Liquid biopsy as a non-invasive approach has been capturing attentions recently. To explore the ability of urine-based liquid biopsy in detecting and monitoring genitourinary tumors, we developed a method based on promoter-targeted DNA methylation of urine sediment DNA. We used samples from a primary bladder cancer cohort (n=40) and a healthy cohort (n=40) to train a model and obtained an integrated area under the curve (AUC) > 0.96 in the 10-fold cross-validation, which demonstrated the ability of our method for detecting bladder cancer from the healthy. We next validated the model with samples from a recurrent cohort (n=21) and a non-recurrent cohort (n=19) and obtained an AUC > 0.91, which demonstrated the ability of our model in monitoring the progress of bladder cancer. Moreover, 80% (4/5) of samples from patients with benign urothelial diseases had been considered to be healthy sample rather than cancer sample, preliminarily demonstrating the potential of distinguishing benign urothelial diseases from cancer. Further analysis basing on multiple-time point sampling revealed that the cancer signal in 80% (4/5) patients had decreased as expected when they achieved the recurrent-free state. All the results suggested that our method is a promising approach for noninvasive detection and prognostic monitoring of bladder cancer.

## One sentence summary

We developed a method for the highly sensitive noninvasive detection and monitoring of bladder cancer based on promoter-targeted DNA methylation of urine sediment DNA.

## Background

Urothelial bladder cancer is a major cause of morbidity and mortality worldwide with approximately 573,000 new cases and 213,000 deaths in 2020 (1), and the incidence of urothelial bladder cancer is higher in men than in women (1). One of the biggest challenges in treating bladder cancer is its high recurrence and metastasis risk. Transurethral resection of bladder tumor (TURBT) is currently the main diagnosis approach and treatment for non-muscle invasive bladder cancer (NMIBC), accompanied by other adjuvant therapies, such as perfusion chemotherapy1 (2). To some extent, adjuvant therapy can reduce the risk of recurrence and metastasis of bladder cancer, but it still cannot completely prevent cancer progression, so regular monitoring of bladder cancer is necessary for the early detection of tumor progression and for prolonging patient survival.

At present, the diagnosis and monitoring of bladder cancer is mainly based on cystoscopy and biopsy, or TURBT, which is invasive, costly and uncomfortable. Urinary cytology is an important auxiliary method for cystoscopy, but its sensitivity is still unsatisfactory, ranging from 21% to 50%, minimal malignant changes can be easily missed (3). Therefore, it is necessary to develop a new monitoring method. Liquid biopsy has gained increasing attention in the field of cancer diagnostics, and urinary markers may serve as an ideal source of “liquid sample” for diagnosis and surveillance for urinary tract tumors. Urine contains tumor cell-free DNA and exfoliated tumor cells and can be obtained easily, regularly and noninvasively (4). In the context of bladder cancer, studies showed urine had better concordance with tumors compared to plasma (5), and it can be sampled in large volumes, allowing routine sampling and improving patient compliance (4).

At present, the potential clinical value of urine exudated cell DNA has been widely explored. TP53 mutations in urine exuded cells from patients with bladder cancer have already been reported 30 years ago (6). Specific mutations in other oncogenes, such as PIK3CA, RAS, FGFR3 and TERT, have also been found in circulating tumor DNA (ctDNA) from bladder cancer patients, which has led to the discovery of an association between the presence of ctDNA mutations in genes from urine samples and disease progression (6–9). Moreover, other common genetic variations in bladder cancer, such as microsatellites, the polymorphic repeat units of 1 to 6 base pairs in length in human DNA, and the loss of heterozygosity on chromosomes 4P, 8P, 9p, 11P and 17p are detected in urinary DNA samples (10–12). In general, the sensitivities of these markers are between 72% and 97%, and the specificity range from 80% to 100% (13, 14). In addition, the role of telomerase in the diagnosis of bladder cancer has been extensively reviewed (15–24), and the sensitivity of telomere repeat expansion protocols allows the enzyme to be detected in exfoliated cells collected in normally excreted urine or bladder irrigation, and telomerase activity can be very easily detected in samples containing 10 to 100 tumor cells. Despite the high sensitivity of telomere repeat amplification protocols, studies have reported a wide variation in detectable activity in urine from bladder cancer patients, ranging from 0% to 86% (25–27).

Altered DNA methylation has been recognized as a crucial cause of cancer development (28). DNA methylation sites tend to cluster in a large number of repeated sequence regions, known as CpG Islands (CGIs) (29). Hypermethylation of CpG islands in the promoter regions of tumor suppressor genes can inhibit the transcription of tumor suppressor genes, leading to the occurrence of tumors (28, 30), DNA methylation has high tumor cell specificity, and its genetic stability makes DNA methylation an ideal marker for cancer diagnosis and prognostic monitoring. Changes in genome-wide DNA methylation patterns are common features in bladder cancer (31–33). Previous studies have shown that changes in DNA methylation events of bladder cancer are reflected in the methylation status of urinary exfoliated cells in non-muscular and muscular invasive bladder cancers, as well as in normal urethral epithelium (31, 34, 35). Therefore, due to its heredity and stability, abnormal DNA methylation in urinary sediment can be used as a potential biomarker for the development of non-invasive urine-based bladder cancer diagnosis and post-treatment monitoring technology (32, 33).

To date, a total of 114 urine methylation markers of primary bladder cancer have been investigated so far, of which 23 primary markers were studied in more than three articles, and the median sensitivity of these biomarkers reached 80%. In addition, 18 individual markers and 8 panels were used for recurrence detection, and the most sensitive biomarker combinations (CFTR, TWIST1 and SALL3; sensitivity 90%) somehow had very low specificity (31%) (36). In addition, there was a large variation in sensitivity and specificity of biomarkers among these studies, which may be due to differences between etiological studies, tumor heterogeneity, technical limitations, or differences in detection sites, highlighting the necessity for additional investigations to determine the sensitivity and specificity of urinary DNA methylation markers for bladder cancer, especially the specificity of DNA methylation markers in the recurrence stage of bladder cancer. Moreover, a major limitation of previous research is that the number of CpG sites contained by DNA methylation biomarker panels is relatively small, and no study could detect all CpG loci in the entire promoter regions, so as to map the methylome of human bladder cancer. To address these issues, in this work, we used a large DNA methylation panel with increased coverage of the promoter regions and developed a specific and sensitive method for diagnosis and recurrence surveillance of bladder cancer with urine sediment DNA.

## Materials and methods

### Study patients and specimens

**Figure 1:**
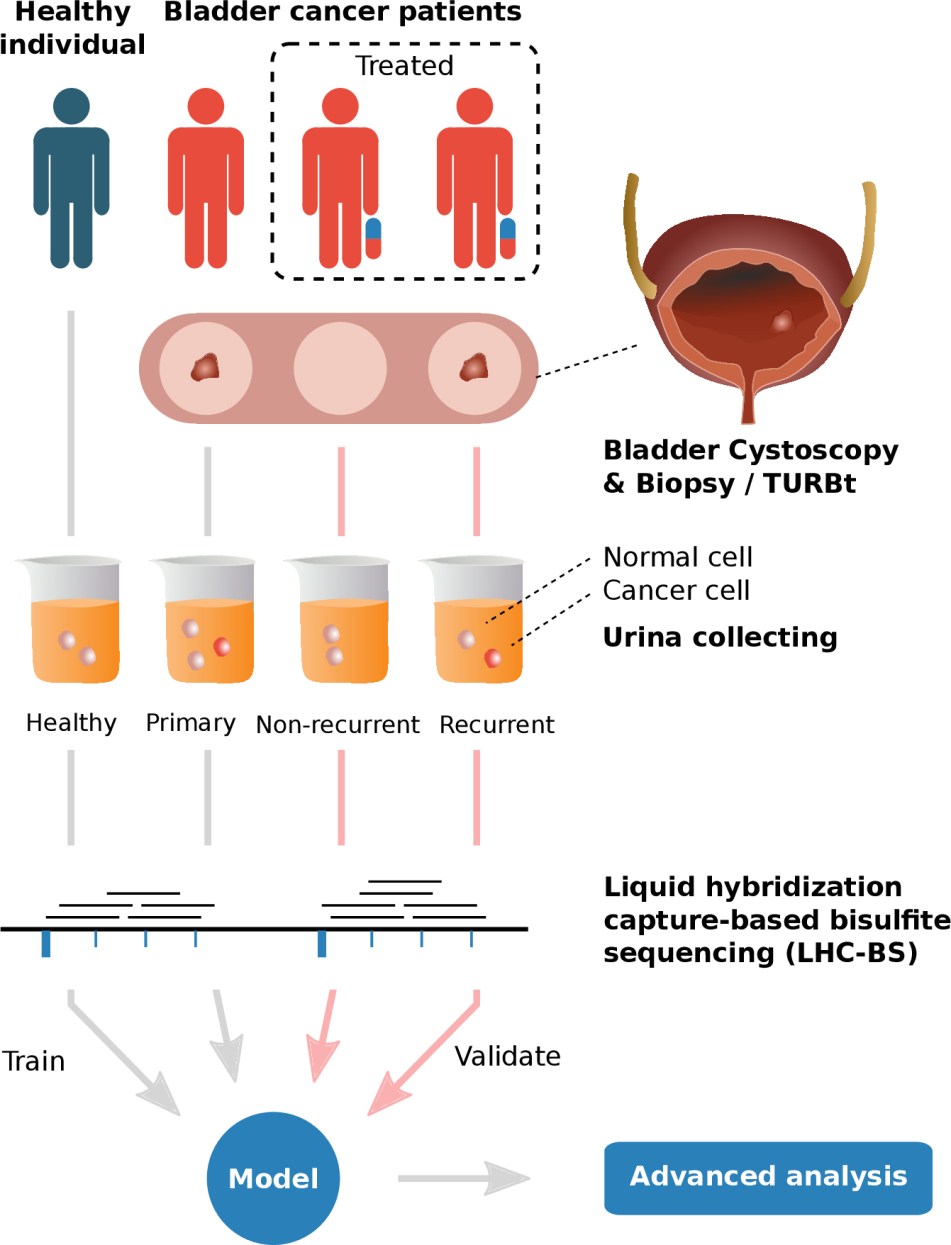
The graphical abstract of analyses performed in this study 1) Urine samples of healthy individuals and different types of bladder cancer patients (primary, recurrent and non-recurrent cohorts, diagnosed with cystoscopy & biopsy/TURBT) were collected and extracted to obtain exfoliated tumor cells for promoter-targeted LHC-BS. 2) Reads of the sequencing data were mapped in target regions and the methylated ratios were counted in frames with fixed number of methylation sites. 3) A model was trained with the methylated data of healthy cohort and primary cohort in the process of 10-fold cross-validation and validated with the data of recurrent & non-recurrent cohort. 4) Advanced analysis, e.g. pathway analysis, had been performed on the patterns of the model.

We collected 80 urine samples from bladder cancer patients, classified into primary cohort (n=40), recurrent cohort(n=21) and non-recurrent cohort (n=19), as well as 45 urine samples from patients with benign (referred to benign cohort, n=5) and healthy (referred to healthy cohort, n=40) donors at the Department of Urology, Peking University Shougang Hospital from November 2017 to December 2019 (**Figure 1**, **Table 1** and **Supplement Table 1**). All patients were diagnosed with bladder cancer by cystoscopy and biopsy, or TURBT. All patients were treated with surgical resection and perfusate chemotherapy using epirubicin hydrochloride or pirarubicin hydrochloride. Urine samples from recurrent and non- recurrent patients were collected before each perfusate chemotherapy, while urine samples of the primary patients were collected before the operation. In addition, clinical and follow-up information of all patients were collected. All experiments were performed in accordance with relevant guidelines and regulations.

**Table 1:** Summaries of clinical characters of different cohorts*.

		Healthy	Primary	Recurrent	Non-recurrent	Benign
Count		40	40	21	19	5
Age	Mean ± SD	55 ± 9	72 ± 12	75 ± 11	68 ± 11	68 ± 17
	*p* value	5.30E-09		0.041		–
Gender	Female	31	8	2	4	1
	Male	9	32	19	15	4
	*p* value	4.30E-07		0.4		–
Smoking**	YES	5	8	3	5	0
	NO	23	32	18	14	5
	*p* value	1		0.44		–
Stage	Ta	0	14	9	0	0
	T1	0	11	4	0	0
	T2	0	10	3	0	0
	T3	0	2	3	0	0
	TIS	0	0	1	0	0
Grade	Low	0	5	1	0	0
	High	0	32	20	0	0

* Six patients provided multiple samples during their treatment progresses, e.g. primary, recurrent and non-recurrent, their clinical information had been used in different cohorts repeatedly; p values were calculated with Fisher’s exact test.

** One patient who quit smoking 20+ years ago was considered to be non-smoking.

### Ethics statement

The study was approved by the institutional review board of Peking University Shougang Hospital (IRBK-2017-053-09). All samples were collected with written informed consent from adult participants, and minors’ informed consent was given by their guardians.

### Sample processing and promoter-targeted liquid hybridization capture-based bisulfite sequencing

Before DNA extraction, 40 mL urine samples were centrifuged at 4°C, 2000 g for 10 minutes to collect the pellet for DNA extraction and urinary sediments were stored at -80° Celsius. DNA of the urine sediments were extracted with TIANamp Micro DNA Kit DP316 (TianGen) per the manufacturer’s instructions. DNA concentrations were measured using a Qubit fluorometer (Invitrogen) to determine DNA input from each isolate. We optimized method based on the published protocol (37), briefly, 500 ng of urine exfoliate DNA was fragmented into approximately 150-200 bp using a sonication system (Covarias). After purification, the fragmented fractions were treated with blunt end repair, 3′adenylation, and 5′-methylcytosine index adapter ligation. The constructed libraries were determined by Qubit (Invitrogen). Then, 250 ng DNA from each of adapter ligated libraries were pooled together for the liquid hybridization capture procedure. After capture program, we then applied bisulfite treatment, and PCR amplification based on described previous protocols (37). Libraries was performed using BGISEQ-500 sequencing platform developed by BGI-Shenzhen in 2015 (38). Promoter-targeted DNA methylation sequencing data that support the findings of this study have been deposited into CNGB Sequence Archive (CNSA) of CNGBdb with accession number CNP0001248 (https://db.cngb.org/cnsa/) (39, 40).

### Data processing

Raw sequencing reads were filtered by SOAPnuke v2.0.7 with parameters ‘-l 5 -q 0.5 -n 0.1 -f AAGTCGGAGGCCAAGCGGTCTTAGGAAGACAA -r AAGTCGGATCGTAGCCATGTCGTTCTGTGAGCCAAGGAGTTG -t 2,0,20,0 -Q 2 -G 2 -T 6 –seqType 0’ (two bases were trimmed off the 5′ end of reads1, while 20 bases were trimmed off the 5′ end of reads2; remove reads that containing adaptor sequence, more than 10% N bases, or more than 50% bases with quality less than 5) (41). Clean reads were mapped to the hg19 genome assembly downloaded from GATK resource bundle (ftp://gsapubftp-anonymous@ftp.broadinstitute.org/bundle/hg19/ucsc.hg19.fasta.gz) by BitMapperBS v1.0.2.3 with default parameters (42). Duplicates were removed by Picard v2.18.27 (http://broadinstitute.github.io/picard/) with parameters ‘REMOVE_DUPLICATES=true’. Quality control were performed using bamdst v 1.0.9 (https://github.com/shiquan/bamdst) (**Supplement Table 2**).

A genome-wide cytosine methylation report was extracted by MethylDackel v0.3.0-3-g084d926 (https://github.com/dpryan79/MethylDackel) with parameters ‘–CHG –CHH –cytosine_report’, every cytosine in any sequence context (CpG, CHH and CHG) on both the plus and minus strands was considered irrespective of whether they were actually covered by any reads in the experiment or not. The capture regions (totally 19,050 regions) were split into non-overlapping 500bp-fixed-length windows. Windows with length less than 500bp were eliminated and totally 120,215 windows were retained for methylation calculation.

**Figure 2:**
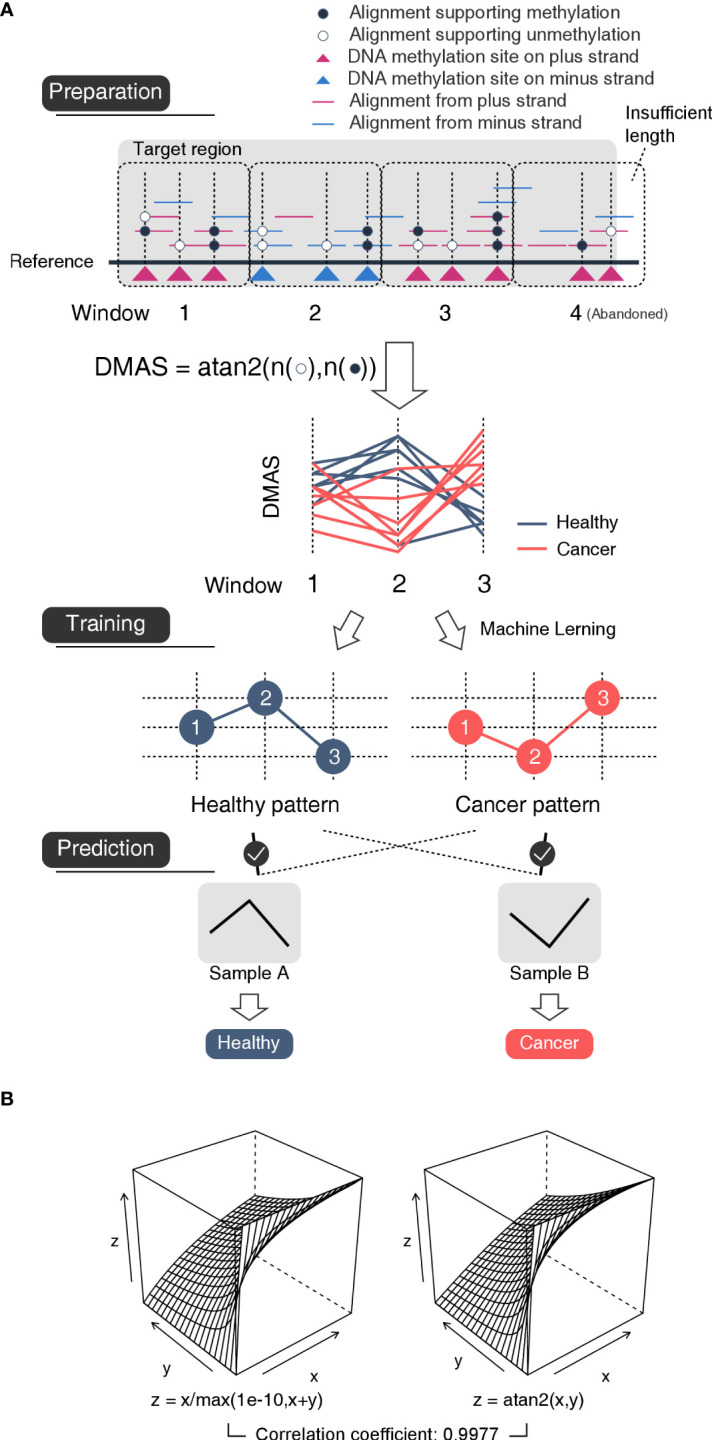
Schematic diagram of the mechanism of the model **(A)** The flowchart of method. Preparation: Each target region was divided into a series of 500bp-length-fixed windows. For each window, the number of unmethylated nucleotides and that of methylated nucleotides were used to calculated an arctangent value DMAS which was inversely proportional to the methylated ratio. In this case, the window #4 was abandoned for insufficient length. Training: Based on the DMASs, a machine learning approach was used to mine the type-specific patterns among neighboring windows. Prediction: A sample would be considered to be the type whose specific patterns appeared in the sample more often. **(B)** In this work, we used DNA methylation angle score (DMAS) instead of DNA methylation rate for the purpose of dealing non-reads-mapped windows. DMAS is highly similar value distribution with DNA methylation rate without extra concern about *x*+*y*=0. Left: DNA methylated ratio is calculated with formula z(x,y)=x/(x+y), where x is the sum of number of alignments supporting unmethylation and y is the sum of number of alignments supporting methylation of all sites in the same window. However, as a dividend, the value of x+y cannot be 0, which could cause problem when a region has no reads mapped, we changed the dividend into max(1e-10,x+y) to make sure its value always above 0. Right: DMAS is calculated with formula z(x,y)=atan2(x,y), where the value of x+y could be 0.

To describe the methylation alteration properly, we put forward the DNA methylation arctangent score (DMAS) which was calculated using the arctangent of unmethylation count and methylation count for each window from each sample by atan2 function in Perl:

*DMAS*(*WIN*_*i*,*j*_)=*atan*2(*unmethyl*(*WIN*_*i*,*j*_), *methyl*(*WIN*_*i*,*j*_)) where *unmethyl*(*WIN*_*i*,*j*_) the sum of number of alignments supporting unmethylation of all sites in window i on sample j, *methyl*(*WIN*_*i*,*j*_) the sum of number of alignments supporting methylation of all sites in window i on sample j, a *DMAS*(*WIN*_*i*,*j*_) value ranges from 0 to $\frac{\pi }{2}$ the DMAS of window i on sample j (**Figure 2A**). The smaller DMAS relates to the higher methylation rate of a window (**Supplement Table 3**). DMAS allows normalization of unmethylation count and methylation count in terms of variability of the sequencing coverage (including non-coverage which results in DMAS value of 0), and exists highly similar value distribution (R=0.9977) with DNA methylation rate (**Figure 2B**).

### Training and validation

With the DMAS data, we trained a bladder cancer detecting model using our previously developed method (43). The machine learning method focuses on identifying and extracting patterns, which are found frequently in one type of sample but may be rare in other types, and uses them as features for cancer prediction. The patterns here, refers to the relative order of windows about their DMASs. For example, three windows WIN_1_, WIN_2_ and WIN_3_ of DMASs from a sample that follow such a relationship DMAS(WIN_2_)> DMAS(WIN_1_)> DMAS(WIN_3_), order (WIN_1_, WIN_2_ and WIN_3_) will be a specific pattern for this sample (**Figure 2A**). We believe such patterns could reflect small changes in methylation under sub-genetic resolution and could provide more details than genetic methylation rates. After structuring a model with those healthy- and BLCA-specific patterns, we validated the model with Recurrent and Non-recurrent cohort. The whole pipeline could be found at https://github.com/HanL233/BLCA_detecting.

### Pathway analysis

We used DAVID (44) an online bioinformatic tool for pathway enrichment analysis after annotating pattern-related genes with ANNOVAR v2018-04-16 (45). The pattern-related gene list was enriched with KEGG pathways by 'Functional Annotation Tool' of DAVID.

## Results

A promoter-targeted liquid hybridization capture-based bisulfite sequencing (LHC-BS) method (33, 39)was applied to profile the promoter methylome of 125 urine samples collected from 80 patients with bladder cancer, 5 patients with benign urothelial diseases and 40 healthy individuals. Urine samples were divided into two groups including the training set and the validation set, the training set consisted of 40 patients with biopsy or TURBT proven primary bladder cancer and 40 healthy donors. The validation set included 21 patients with recurrent bladder cancer, and 19 patients without recurrence undergoing surveillance. Characteristics of patients from each group are listed in **Supplemental Table 1**. The healthy cohort was composed of patients with younger ages comparing to the primary cohort (T test p=5.3e-09), and the non-recurrent cohort is the younger cohort comparing to the recurrent cohort (T test p=0.041) (**Table 1**).

### The establishment of a model for bladder cancer detection based on urine sediments DNA methylation assay

**Figure 3:**
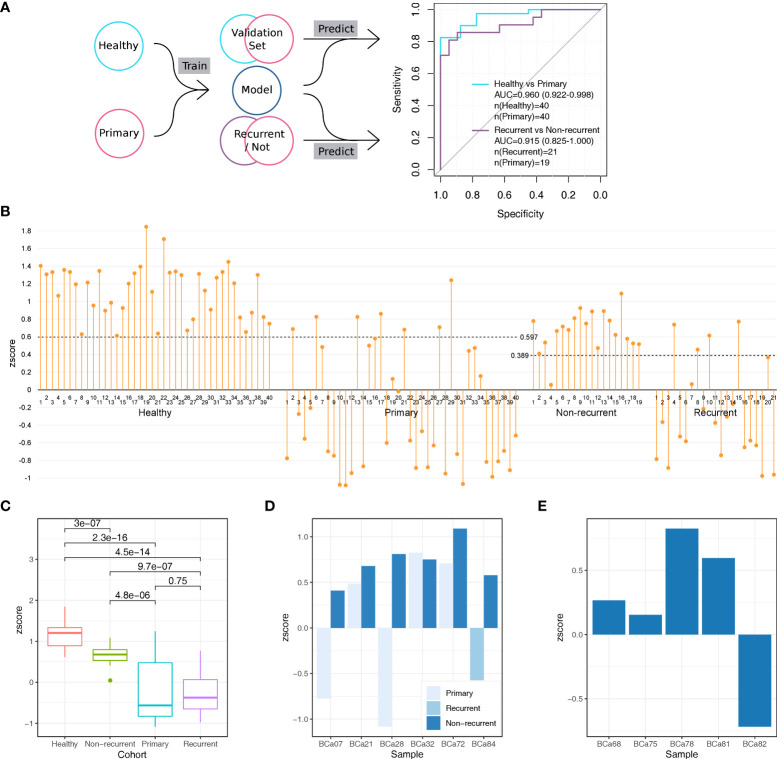
The establishment and validation of a model for bladder cancer detection based on urine sediments DNA methylation assay. **(A)** We performed the 10-fold cross-validation on the healthy cohort and primary cohort to distinguish samples from the two cohorts. We calculated an AUC of 0.960 (95%CI: 0.922-0.998) based on all validation set samples. We test the 10 models trained during the 10-fold cross-validation to distinguish samples from the recurrent cohort and the non-current cohort, which provided 10 zscores for each sample. For each sample, we summed its 10 scores as its integrated scores, with which we obtained an AUC of 0.915 (95%CI: 0.825-1.000). **(B)** The zscore distributions of samples from different cohorts. The cutoff values were provided by function roc in R package pROC. **(C)** The differences in zscore among different cohorts. All p values were calculated with the Wilcox test which was the default opinion for R package ggpubr. **(D)** Comparisons in zscore between samples provided by the same patient (6 patients in total) **(E)** The distributions of zscores of 5 individuals with benign urothelial diseases.

We trained a model with the DMAS data of the healthy and the primary cohort using an algorithm developed previously (46) with the form of 10-fold cross-validation. Samples were divided into 10 equal subsets randomly. For each round of the cross-validation, samples of one subset were used as the validation set, and the rest were used as the training set. After 10 rounds of cross-validation, every sample had been used as a validation sample at least once. Each validation sample would be scored by the model to determine its types, as the results, every sample in the healthy cohort and the primary cohort got its score. The sum of weights of healthy-specific patterns was referred as the sample’s H-zscore and that of the cancer-specific patterns was referred as its C-zscore. The H-zscores and C-zscores of samples from the same subsets were normalized with their D-zscores (z scores), which is the result of H-zscore minus C-zscore (**Supplement Table 1**). With the z scores, we obtained an integrated AUC of 0.960 (95%CI: 0.922-0.998, **Figure 3A**), which demonstrated our assay’s ability in differentiating bladder cancer patients from healthy individuals non-invasively.

### The validation of the model in bladder cancer recurrence surveillance and benign cancer detection

To demonstrate whether the aforementioned model could be used in surveillance for bladder cancer recurrence, we applied it on the recurrent and non-recurrent cohorts (**Figure 3A**). We used the 10 trained models obtained from the previous 10-fold cross-validation to score each sample, as the results, each sample would obtain 10 zscores. We took the mean of 10 zscores of each sample as its final zscore (**Supplement Table 1**), based on which we obtained an AUC of 0.915 (95%CI: 0.825-1.000). The high AUC indicated that our method’s ability in monitoring bladder cancer non-invasively.

We observed significant differences in zscore among cohorts (**Figures 3B, C**). Notably, for the healthy cohort, its zscores were not only higher than that of the primary cohort (Wilcox test p=2.3-16) and the recurrent cohort (Wilcox test p=4.5e-14) but also higher than that of non-recurrent cohort (Wilcox test p=3e-7). As the lower zscore indicated higher risk of bladder cancer, this result suggested that the urine samples of non-recurrent cohort still to some extent remain cancerous, although non-recurrent cohort’s zscores were higher than the primary cohort (Wilcox test p=4.8e-6) and recurrent cohort (Wilcox test p=9.7e-7).

We next checked how the zscores changed over the treatment. There were 6 bladder patients donated more than one urine sample during the treatment which provided an opportunity to analyze how the cancer signal changed over this period. All 6 patients achieved the progress-free (non-recurrent) stage after treatment and 5 of them show zscores higher than their previous stage, primary or recurrent (**Figure 3D**). The results indicated our method is sensitivity to track the cancer signal level in urine sample.

In addition, we also validated the model with the benign cohort (**Figure 3E**). There were 5 individuals with benign urothelial diseases in the benign cohort. We used the same steps which used in the validation with recurrent and non-recurrent cohort to score samples from the benign cohort. As the results, the zscores of 4/5 individuals were above 0, which means the model considered them could come from healthy donors rather than bladder cancer patients. This suggested that our method reached high accuracy (80%) in detecting benign urothelial diseases from bladder cancer.

### Analysis on potential confounding factors of the model

**Figure 4:**
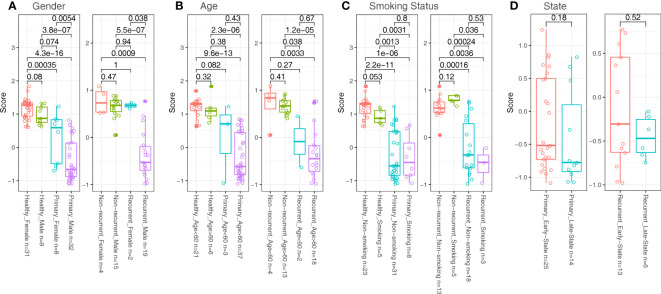
Comparisons in zscore between samples divided with clinical factors in different cohorts. **(A)** Samples were divided with gender factors (Female vs Male). **(B)** Samples were divided with age factors (<60 vs >60). **(C)** Samples were divided with smoking status (Non-smoking vs smoking). **(D)** Samples were divided with state factors (Ta, T1 as Early States; T2, T3 as Late States; for patients only).

We analyzed the potential effect of clinical factors on model’s performance. We divided samples from the same cohort into two groups with incompatible clinical conditions, such as gender (female vs male, **Figure 4A**), age (<60 vs >60, **Figure 4B**), smoking status (non-smoking vs smoking) and stages (early states (Ta, T1) vs late states (T2, T3), for bladder cancer patients only, **Figures 4C, D**) and measure the differences in zscore between the two groups. The results suggested that factors like age, smoking status, stage unlikely affected the model’s performance (Wilcox test p value between two groups from the same cohort were all non-significant (>0.1). However, for the primary and the recurrent cohort, there were significant differences in zscores between the two groups divided by gender factor (Wilcox test p<0.05, **Figure 4A**). Of note, the sample size is extremely small in the recurrent female cohort (n=2), so a definitive conclusion may not be drawn for this cohort.

### Pathways analysis on patterns composing the model

We explored the biological meanings of patterns composing the models by annotating the regions where the patterns located. We focused on 1471 genes (for patterns which located in intergenic region, we chose the nearest gene from its upstream/downstream genes as its corresponding gene). With further pathway analysis, we found two related pathways (hsa04020: Calcium signaling pathway, Modified Fisher Exact p=3.53E-05, Bonferroni=0.01; hsa04012: ErbB signaling pathway, Modified Fisher Exact p=1.39E-04, Bonferroni=0.04).

## Discussion

LHC-BS approach is an efficient and reliable analytical platform (37, 47). In this study, we further optimized the technology of LHC-BS and performed promoter methylation analysis on urine exfoliated cell DNA of 125 urine samples. We carried out the largest genome-wide DNA methylation screening of bladder cancer so far, defined a gene panel covering 91.8% of the promoter regions, which could detect 1.86 million CpG sites. With the results from the optimized panel, we first trained a model with urine samples from the primary and the healthy cohort, which obtained an AUC > 0.96, and then validated it with samples of the recurrent cohort and the non-recurrent cohort, with obtained an AUC of 0.92.

We put forward the concept of DMAS to describe methylation states. Methylation ratio is calculated by dividing the number of total nucleotides covering target sites with the number of methylated nucleotides. Obviously, the number of total nucleotides couldn’t be zero, which requires extra process to deal with sites with no reads covered. DMAS correlated highly with methylation ratio avoiding concern of the mentioned sites. With the DMAS data, we trained models using previous machine learning method (42, 43). Our method focuses on the DMAS patterns formed with adjacent bins instead of focusing single DMAS value alteration, which could produce more robust models in theory. Our method was developed basing on the reads number within bins, and it worked surprisingly well in another different kind of data and strengthened our confidence to explore its potential in cancer detecting and monitoring.

Many studies have explored the use of urine exfoliates cellular DNA as a biomarker for the diagnosis or prognosis monitoring of bladder cancer (4), showing promise with sensitivity to detect bladder cancer between 67%~100% based on methylation assays, but none of them have progressed into clinical practice (48–56). The quantity of loci that can be included within a panel to analyze methylation and the low amount of DNA that can be extracted from urinary exfoliated cells have been important limiting factors for methylation detection development. In the past two years, several research groups have combined next-generation bisulfite sequencing with target genome capture technology, to overcome these issues, allowing a large number of epigenetic biomarkers from a single sample. Specifically, Andrew Feber et al. evaluated DNA in the cellular fraction of urine samples, achieving 98% sensitivity and 97% specificity in identifying bladder cancer at primary diagnosis (57). However, this assay mainly answered the specific question about the primary diagnosis of bladder cancer in hematuria patients and has not been tested in the recurrence setting. As reported in previous articles, urinary biomarkers in detecting recurrent bladder cancer have generally fared less well than in the primary diagnosis setting with sensitivities ranging from 46–74% (32, 58–60). Our promoter methylation detection panel combined with second-generation sequencing, presented in this study, improved the sensitivity of recurrence monitoring analysis. Additionally, for the first time, we compared the methylation signals of the healthy cohort and the non-recurrent cohort. Interestingly, although the methylation signals of the non-recurrent cohort were more similar to those in the healthy cohort than to bladder cancer patients, there were still significant differences between the non-recurrent cohort and the healthy cohort (**Figure 3C**), suggesting that the non-recurrent cohort still retained tumor signals despite no progress in their disease.

Previous research has shown robust urinary test for the detection of bladder cancer; however, few have explored the possibility of differentiating cancer from benign genitourinary diseases caused by inflammation. We here compared methylation signals between patients with benign genitourinary diseases, and those with bladder cancer (**Figure 3E**). In our model, 80% of patients with benign diseases had been distinguished from cancer samples, despite tumor and inflammatory tissues have similar changes in the microenvironment (61). Numerous studies have shown that epigenetic alterations, such as DNA methylation, play a crucial role in carcinogenesis, especially in inflammation-related cancers (62) and both inflammation and oncogenesis cause similar alterations in methylation signals, which potentially increased difficulty in distinguishing cancer and inflammatory diseases by methylation signals from urine DNA. Despite a small number of inflammatory samples used in this study, we preliminarily demonstrated the potential of our assay in differentiating cancer from inflammation.

As for the influence of clinical factors on model’s performance, we found that age, smoking status and clinical stages have no significant influence on the performance of our model. However, the gender factor had shown significant effect on the performance, especially in the primary cohort. This may be due to the gender-related methylation difference globally, and it is known that the incidence of bladder cancer is higher in men than in women (1). Although sex differences in DNA methylation levels in human urine have not yet been studied, it has been shown in some cell types and human tissues, such as heart muscle, liver and blood (63–67). Our results provide a reference for the subsequent studies on the correlation between DNA methylation and gender in urine. Sex differences in DNA methylation may explain the different risk of bladder cancer in men and women, and further studies are needed to explain these issues.

Calcium signaling plays a crucial regulatory role in the invasion and migration of tumor cells by activating calcium-binding proteins or other effector proteins in various signaling pathways (68). Through enrichment analysis of epigenetically silenced genes signaling pathways and gene interaction network analysis, it was found that most calcium signaling pathways showed low expressed genes with hypermethylation. In addition, these genes whose expression regulated by DNA methylation are mostly located at key nodes of the calcium signaling pathway, including the Ca^2+^‐Na^+^ exchange and G-protein-coupled receptor, which control the calcium flow (69). ErbB signaling pathway also plays an important role in the occurrence and development of cancers by the transduction of mitogenic signals (70). Several researches reported on the methylation of ErbB signaling network genes in various tumors (71–73). Epigenetic changes, such as methylation of CpG islands in the promoter region, result in significant silencing of gene expression (74).

Limitations of our study mainly include small sample sizes and a lack of case-control design for patients in the primary and surveillance groups. Due to the limited number of samples, especially for benign bladder tumors, biological variability between samples could be magnified, affecting the accuracy of our model statistically and the potential of urine exocytic cells for early prediction of bladder cancer. To overcome these limitations, further studies with larger sample sizes that are sequenced on a whole genome-wide methylation level and possibly involved both inflammatory and early cancerous samples will be needed to evaluate the sensitivity and specificity of our model in predicting early-stage primary bladder cancer and in distinguishing early-stage bladder tumors from benign or from other bladder diseases.

In conclusion, our results suggest that the method we developed could provide a high sensitivity in non-invasive detecting and monitoring bladder cancer, which could reduce the frequency of costly invasive cystoscopy. Despite clinical importance of this powerful model, cautious interpretation and further studies are warranted to improve its performance and expend its application.

## Data availability statement

The datasets presented in this study can be found in online repositories. The names of the repository/repositories and accession number(s) can be found below: CNGB Sequence Archive (CNSA) of CNGBdb, CNP0001248.

## Author contributions

NNL, HL and JY designed the study. LW, LZ and XL contributed to sample acquisition and clinical information collection. NNL, HJL and TL contributed to experiment performance. HL, FL and QY contributed to data collection, analysis and interpretation. KW and NCL supervised the study. NNL, HL, FL and CL wrote the manuscript. All authors reviewed the paper.

## Funding

This work was supported by the Guangdong Provincial Key Laboratory of Human Disease Genomics (2020B1212070028).

## Conflict of interest

The authors declare that the research was conducted in the absence of any commercial or financial relationships that could be construed as a potential conflict of interest.

## Publisher’s note

All claims expressed in this article are solely those of the authors and do not necessarily represent those of their affiliated organizations, or those of the publisher, the editors and the reviewers. Any product that may be evaluated in this article, or claim that may be made by its manufacturer, is not guaranteed or endorsed by the publisher.
